# Implications of Localized ST Depression in a Vascular Territory and Altered Precordial T-Wave Balance in Ischemic Heart Disease

**DOI:** 10.7759/cureus.8580

**Published:** 2020-06-12

**Authors:** Himal Kharel, Nishan B Pokhrel, Biraj Pokhrel, Parikshit Chapagain, Chandra M Poudel

**Affiliations:** 1 Internal Medicine, Tribhuvan University, Institute of Medicine, Maharajgunj Medical Campus, Kathmandu, NPL; 2 Cardiology, Tribhuvan University, Institute of Medicine, Maharajgunj Medical Campus, Manamohan Cardiothoraccic Vascular and Transplant Centre, Kathmandu, NPL

**Keywords:** acute coronary syndrome, electrocardiogram, st elevation myocardial infarction, non-st elevation myocardial infraction

## Abstract

The incidence of acute coronary syndrome (ACS) is rising globally. Electrocardiography is still one of the best diagnostic modalities for it. Although some of the ECG changes of ACS are well known among medical practitioners, there are a handful of ECG changes that do not get the recognition they deserve. Among these are localized ST-segment depressions in a vascular territory and altered precordial T-wave balance. The urgency of management varies among the various subtypes of ACS, especially in low resource settings. ST-segment depression localized to a vascular territory is a sign of ST-elevation myocardial infarction (MI) in the reciprocal lead which may not always be evident and hence, requires emergent reperfusion therapy. On the other hand, altered precordial T-wave balance (T1 > T6, T-wave in V1 > 1.5 mm and upright T-wave in V1) may be predictive of significant coronary artery disease (CAD).

## Introduction and background

In patients with myocardial infarction (MI), the ST depression localized to a vascular territory in electrocardiography is a frequent, yet overlooked sign. Although localized ST depression in anterior precordial leads is widely recognized as a sign of posterior ST-elevation myocardial infarction (STEMI), the same is not true for localized ST depressions in other vascular territories. In that case, the ST depression may be mistaken for non-ST elevation myocardial infarction (NSTEMI) or unstable angina. This may delay emergent reperfusion therapy. Similarly, the loss of precordial T-wave balance is also a valuable sign when it comes to a patient with chest pain. These signs should be specifically observed as they are easily overlooked if not given due attention. Welch et al. have found that normal and non-specific electrocardiograms have combined rates of in-hospital mortality and life-threatening adverse events of 19.2% and 27.5%, respectively [1]. The main aim of this article is to provide a detailed overview of the pathophysiology behind the occurrence of these signs and show how they can be useful in real-life scenarios.

## Review

Localized ST depression in a vascular territory: A subtle sign of transmural infarction and the need for emergent reperfusion therapy

Mechanism of ST-Segment Changes

Ischemia leads to an increase in extracellular potassium cation (K^+^). This is secondary to K^+^ efflux from ATP sensitive K^+^ channels (K_ATP_) that become activated due to low ATP [2]. This accumulation of extracellular K^+^ is considered to be an important mechanism for ST changes in the setting of ischemia. The role of K_ATP_ has been supported by the absence of ST elevation in mice with homozygous knockout (KO) of the Kir6.2 gene which encodes the pore-forming subunit of cardiac surface K_ATP_ channels and the attenuation of ST elevation in people taking sulfonylureas [3,4].

ST-segment elevation: ST-segment is typically at the same voltage as the T-P segment. During diastole, accumulated extracellular K^+ ^at the baseline will produce a resting voltage difference between injured and non-injured areas of the myocardium. This will result in depression of the resting T-P segment which will result in apparent "ST elevation" [5]. In addition to this, the action of K_ATP_ channels will cause earlier repolarization of epicardial tissue compared to myocardial and endocardial tissues because epicardial cells have a greater response to low ATP due either to a larger number of K_ATP_ channels or channels that are more sensitive to decreased levels of ATP [6]. Earlier repolarization of the epicardium and thus the resulting increase of transmural voltage gradient during ischemia will be associated with ST elevation.

ST-segment depression: The pathophysiology of ST depression is still not clear. The most simplistic model is the dipole hypothesis proposed by Wilson et al. [7]. It states that the ischemic area of the myocardium which is situated in the sub-endocardial region acts as the positive pole, while the non-ischemic area of myocardium acts as the negative pole. The ventricular surface and the precordium over the ischemic region face the negative pole of the dipole, leading to ST depression. This hypothesis was discarded because of its inability to explain the clinical difficulty in localizing ST depressions. Kilpatrick et al. hypothesized that ST depression on ECG was due to the current of injury flowing from an endocardial ischemic region to the outside of the heart through the great vessels and atria [8]. This was suggested to be the cause of the non-localizing nature of ST depression. This hypothesis was challenged in the most accepted theory by Li et al. who reported an increase in the magnitude of ST depression when the aforementioned paths were interrupted in sheep heart, wherein that the lateral boundary, which is perpendicular to the endocardium and in between ischemic and non-ischemic tissue, is sharp and distinct, whereas the boundary parallel to the endocardium is not so distinct due to unknown reasons [9]. The sharpness of demarcation between the healthy and ischemic tissues is directly proportional to the potential difference and hence the current is generated. So, the ST depression on epicardium is thought to reflect the position of lateral boundary of the ischemic region which is typically shared between vascular territories. This was suggested to be the reason for the non-localizing nature of ST depression.

Clinical Implications

Localized ST depression is usually a marker of STEMI on reciprocal leads which may not always be evident. This is especially true in high lateral leads where low voltages lead to potential ST elevations not meeting the arbitrarily defined criteria for ST elevation.

**Figure 1:**
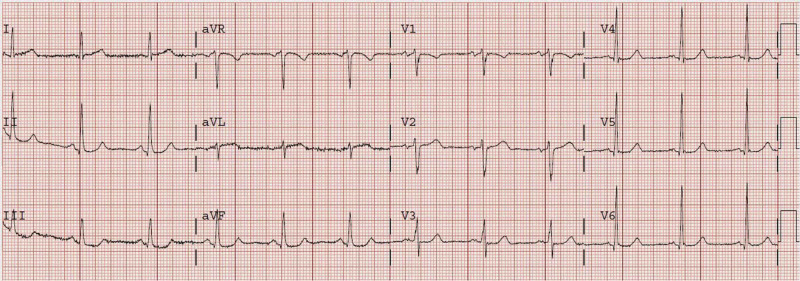
The ECG of a 72-year-old male showing ST depression in leads III and aVF as well as less than 1 mm of ST elevation in aVL Image adapted from Dr. Smith’s ECG Blog [10].

**Figure 2:**
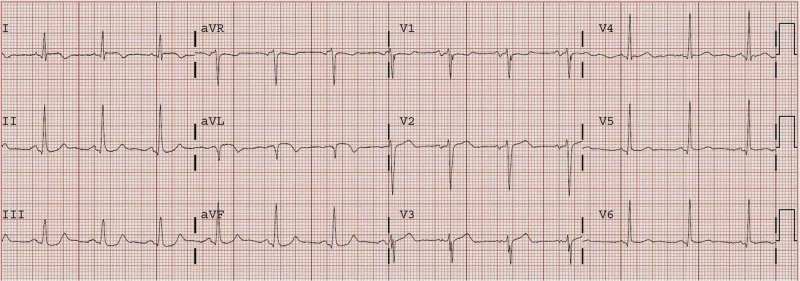
Post-catheterization ECG of the same patient from Figure 1 showing a new Q-wave with T-wave inversion in lead aVL Image adapted from Dr. Smith’s ECG Blog [10].

An ECG of a 72-year-old male with a history of coronary artery bypass graft (CABG) who presented after 5.5 hours of sudden onset constant and substernal chest pressure is presented in Figure 1 [10]. It shows ST depression in leads III and aVF. The initial troponin was 0.82 ng/mL. Unfortunately, the findings were not recognized. Cardiac catheterization was performed only on the following day which showed that the obtuse marginal artery had 90% stenosis with thrombolysis in myocardial infarction (TIMI) I-II flow. Post-catheterization ECG showed a new Q-wave with T-wave inversion in aVL (Figure 2) [10]. The peak troponin I was 8.6 ng/mL, and a new wall motion abnormality was detected in echocardiography. This was, in fact, a missed completed high lateral MI.

New-onset upright T-wave in lead V1: A sign of significant coronary artery disease (CAD)

Mechanism of Formation of T-wave

T-wave is generated due to ventricular repolarization which proceeds from epicardium to endocardium [5]. This is counter-intuitive as the epicardium is the last to depolarize. But due to the shorter duration of the action potential in epicardial cells, it is the first to repolarize. Unlike depolarization, which occurs due to conduction, repolarization occurs through synchronization. This is responsible for the longer duration of the T-wave compared with the QRS complex [11]. The normal direction of the T-wave in the precordial leads generally follows the direction of the QRS complex, but in the anterior leads (V2-V4) an upright T-wave can be observed even in the presence of predominantly negative QRS complex [5]. The T-wave is normally inverted in lead V1. The electric vectors of the heart are such that if positive T-waves are present in lead V1 (which is relatively uncommon), they are usually smaller in amplitude than the T-wave in lead V6. This is called normal precordial T-wave balance. As the T-wave is normally inverted in lead V1, the presence of an upright T-wave in V1 may be abnormal. This finding has been named 'new upright T-wave in V1' (NUTV1) and must be compared with previous ECGs. NUTV1 is also seen in left ventricular hypertrophy, left bundle branch block, and lead displacement.

Clinical Implications

The absence of normal precordial T-wave balance is one of the subtle signs of significant CAD (coronary artery stenosis >50%) in ECG. Studies have shown that the loss of precordial T-wave balance (T1 > T6), T-wave in V1 > 1.5 mm, and upright T-wave in V1 have been associated with significant CAD [12-16]. The reason for this is unclear and needs further research. The summary of the findings of the above studies is presented in Table 1.

**Table 1:** Summary of various related studies on the significance of an upright T-wave in V1 and TV1 > TV6 OR: odds ratio; CI: confidence interval

Study	Findings
Barthwal et al. [12]	TV1 > TV6 sign has sensitivity, specificity, and false positivity of 72.9%, 84.4%, and 15.6% respectively, for significant CAD.
Manno et al. [13]	Upright T-wave in V1 was present in 184 of 218 patients who had >75% occlusion of coronary arteries more frequently on the left circumflex artery.
Nalbantgil et al. [14]	TV1 > TV6 had a sensitivity of 16.1%, the specificity of 95.6%, and accuracy of 94.2% for significant coronary artery stenosis.
Amirzadegan et al. [15]	Upright T-wave in V1 > 1.5 mm had OR = 2.38 (1.78 - 3.12) for coronary artery stenosis of greater than 50%.
Stankovic et al. [16]	Upright TV1 was an independent predictor of significant CAD (OR 4.249, 95% CI 1.594 - 11.328).

## Conclusions

The presence of ST depression localized to a vascular territory may be secondary to reciprocal STEMI, and may indicate the need for emergent therapeutic intervention. In the absence of knowledge about localized ST depression in a vascular territory in cases of non-STEMI, such cases might be acted upon late resulting in increased morbidity and mortality. Similarly, altered precordial T-wave balance (T1 > T6, T-wave in V1 > 1.5 mm and upright T-wave in V1) may be predictive of significant CAD. Further research is still needed in this regard.
